# Metabolic Syndrome, Sleep Quality and Lung Function in Persons With Schizophrenia: A Cross-Sectional Study

**DOI:** 10.1192/bjo.2024.199

**Published:** 2024-08-01

**Authors:** Dinesh M, Dinesh Kataria, Sunita Mondal, Shiv Prasad, Sajjadur Rehman

**Affiliations:** 1 All India Institute of Medical Sciences, New Delhi, India; 2 Lady Hardinge Medical College, New Delhi, India

## Abstract

**Aims:**

Persons with schizophrenia typically have a 20% shorter lifespan and mortality rates two times higher than the general population. More than 2/3 of this is due to different forms of physical diseases, like cardiovascular and metabolic syndrome. Systematic meta-analyses and various studies in schizophrenic patients revealed the prevalence of metabolic syndrome to range from 11 to 69%, poor sleep quality 30% to 80%, and impaired lung function ~30%. Both in the general population and in persons with schizophrenia, poor sleep quality and impaired lung function are associated with a heightened risk of metabolic and cardiovascular diseases. Hence, this study aimed to look for the magnitude of metabolic syndrome, poor sleep quality, and impaired lung function, and any association among them, if proven, may be helpful in better management.

**Methods:**

We included sixty cooperative patients through purposive sampling with an age range of 18 to 65 years, meeting the DSM–5 criteria for schizophrenia, and excluded patients with co-morbid substance use disorder except for smokeless tobacco and caffeine. Harmonized criteria were used to diagnose metabolic syndrome; the Pittsburgh Sleep Quality Index (PSQI) for sleep quality and lung function was interpreted as per the Spirometry for Health Care Providers, Global Initiative for Chronic Obstructive Lung Disease.

**Results:**

55% were found to have metabolic syndrome. Poor sleep quality (PSQI > 5) was found in 60% of cases, with the most common sleep abnormality being increased sleep latency (95%). Restrictive Lung Dysfunction (RLD) was found in 46.7% of cases. 66.7% of the participants with metabolic syndrome had RLD, whereas only 22.2% without metabolic syndrome had RLD. The difference was statistically significant. No statistically significant difference was found between metabolic syndrome and sleep quality or sleep quality and RLD.

**Conclusion:**

From the results obtained, it is clear that the prevalence of metabolic syndrome in people with schizophrenia is twice that of the general population, which also contributes to their increased mortality. Thereby, early identification of metabolic disturbances and correcting poor sleep quality and impaired lung function that are associated with an increased risk of metabolic syndrome will lead to increased life expectancy and a decrease in the mortality rate. Since lung function is studied in only a very few studies all over the world and ours being a novel approach in India showing significant association, it needs to be replicated in a larger sample size.

